# Transition to Skilled Birth Attendance: Is There a Future Role for Trained Traditional Birth Attendants?

**Published:** 2006-12

**Authors:** Lynn M. Sibley, Theresa Ann Sipe

**Affiliations:** ^1^ Center for Research on Maternal and Newborn Survival, Emory University, 1520 Clifton Road, Suite 436, Atlanta, GA 30322, USA; ^2^ Rollins School of Public Health, Behavioral Science and Health Education, Emory University, 1518 Clifton Road, Atlanta, GA 30322

**Keywords:** Traditional birth attendants, Skilled birth attendants, Meta-analysis, Maternal mortality, Perinatal mortality, Training

## Abstract

A brief history of training of traditional birth attendants (TBAs), summary of evidence for effectiveness of TBA training, and consideration of the future role of trained TBAs in an environment that emphasizes transition to skilled birth attendance are provided. Evidence of the effectiveness of TBA training, based on 60 studies and standard meta-analytic procedures, includes moderate-to-large improvements in behaviours of TBAs relating to selected intrapartum and postnatal care practices, small significant increases in women's use of antenatal care and emergency obstetric care, and small significant decreases in perinatal mortality and neonatal mortality due to birth asphyxia and pneumonia. Such findings are consistent with the historical focus of TBA training on extending the reach of primary healthcare and a few programmes that have included home-based management of complications of births and the newborns, such as birth asphyxia and pneumonia. Evidence suggests that, in settings characterized by high mortality and weak health systems, trained TBAs can contribute to the Millennium Development Goal 4—a two-thirds reduction in the rate of mortality of children aged less than 14 years by 2015—through participation in key evidence-based interventions.

## INTRODUCTION

Every year, an estimated four million babies die in the first four weeks of life, and a similar number of babies are stillborn. Moreover, half a million women die from pregnancy-related causes. Most of these deaths occur at home during the first postnatal or postpartum week, especially within the first 24 hours of birth. Most occur in developing countries with weak or failing health systems, with South Asia and sub-Saharan Africa being especially hard-hit (1–3). For more than three decades, the World Health Organization (WHO) and other agencies of the United Nations promoted training of traditional birth attendants (TBAs) as a global public-health strategy to reduce this tragic loss of life (4). Lack of evidence to demonstrate that trained TBAs can reduce maternal mortality led to controversy over their training in relation to safe motherhood and a policy shift to skilled birth attendance (5–7). In this paper, we provided a brief history of TBA training, reviewed evidence of the effectiveness of TBA training, and considered the future role of trained TBAs in an environment that emphasizes the transition to skilled birth attendance.

Although TBAs have been trained since the late 1800s, important milestones over the last century illustrate the shifting policies on TBA training as a global public-health strategy. Credit for the first formal training programme is usually given to a British missionary midwife, Miss M.E. Wolfe, working in Sudan in 1921 (8, 9). The Inter-Governmental Conference of Far-Eastern countries, held in Bangkok in 1937, called for the integration of TBAs into rural health programmes. By 1952, the United Nations Children's Fund (UNICEF) began to supply trained TBAs with delivery-kits. The goal of these early programmes was to improve perinatal healthcare (8). Nearly 20 years later, interest in primary healthcare and in traditional medicine in relation to primary healthcare had grown to the extent that the UNICEF and WHO sponsored a technical consultation on TBA training. By the time of the 1978 Alma Ata Declaration, the WHO was fully in support of training TBAs to extend the reach of primary healthcare services. At that time, the WHO recommended that trained TBAs work side-by-side in ‘articulation’ with the modern health system, so that the informal traditional and formal modern health systems could presumably co-exist without conflict. The success of the WHO's encouragement can be measured by the rapid increase in the number of countries undertaking TBA training. For example, in 1972, only 20 countries had TBA training programmes. It is now estimated that 85% of developing countries have some form of TBA training (8). With the advent of the safe motherhood initiative and without evidence to show that the risk approach and trained TBAs can reduce maternal mortality, there has been a gradual waning of enthusiasm for TBAs. In 1992, the WHO emphasized that, if TBAs were going to contribute to safe motherhood, they must be ‘integrated’ into the modern health system through training, supervision, and technical support (4). However, by 1997, the WHO and many safe motherhood advocates turned from TBA training to promote skilled birth attendance for all (8), most recently calling for a ‘new’ and ‘expanded role’ for TBAs, where TBAs act as ‘link workers’ to skilled birth attendants rather than as primary care providers (10, 11).

The broad goals of TBA training programmes are to reduce maternal and child mortality and morbidity and to improve the reproductive health of women. The objectives include: enhancing the linkages between the modern health system and community, increasing the number of TBA-attended births, and improving the skills and stature of TBAs (4). Training programmes vary, however, in addressing these objectives. For example, individuals, non-governmental organizations, and missions have trained TBAs through the private sector and also through local, state and national government, and international agencies have trained them through the public sector. Training programmes may last from several days to several months and may include clinical practice at a health facility, follow-up supervision, and continuing education (12).

The content of curricula of TBA training also varies. Most TBAs have been trained to upgrade their skills so as to be able to perform safe deliveries. Consistent with the emphasis on extending the reach of primary healthcare, many TBAs have also been trained to take on the expanded functions of prevention, screening, and referral (12, 13). Very few programmes have included content on initial response and stabilization of maternal and newborn complications (e.g. resuscitation of newborns or detection and management of sepsis), and content that would be very useful to those who are confronted with emergencies and that might save lives (13).

The answer to the question: is TBA training effective? depends on the objectives and content of a particular training programme and the outcomes measured. From 1997 to 2002, we conducted a meta-analysis of the available published and unpublished studies on the effectiveness of TBA training. We updated this review for selected topics in 2003 and 2004 (13–15). The goal of the meta-analysis was to provide information that would inform policy decisions about future TBA training and about the needs of evaluation research. Specific objectives were (a) to describe the effect of training on TBA and maternal attributes, such as knowledge and behaviour, (b) to determine the impact on pregnancy outcomes, and (c) to describe the quality of the literature on evaluation.

## MATERIALS AND METHODS

Details of materials, methods, and statistical analyses have been described elsewhere (13–15). Briefly, a broad search yielded nearly 1,200 documents, of which 60 studies met the following eligibility criteria: the intervention was TBA training; intervention group data were derived from trained TBAs (reference to intervention), and mothers and neonates whose care was provided by trained TBAs or who were living in areas where more than 50% of births were attended by trained TBAs (a proxy for exposure); comparison group data were available; dependent measures were related to knowledge, attitudes, behaviour, or maternal and peri-neonatal health outcomes; documents were in English and published during January 1970–June 1999; research design was either experimental or quasi-experimental; and data were sufficient to calculate an effect size. The sample of studies, representing three regions of the world—Asia, Africa, and Latin America/Caribbean—contained 1,695 outcomes. TBA and maternal attributes comprised 95% of the outcomes, and pregnancy outcomes comprised the remainder. The attribute outcomes were independently sorted into 24 maternal and child health content areas for sub-group analysis. In 2002, we repeated the search for studies having outcomes relating to antenatal care and referral for obstetric complications. In 2004, we repeated the search for studies having outcomes relating to safe delivery and newborn-care practices. Altogether, these update searches yielded two new studies plus three documents that were more recent versions of studies already included in the original sample.

Most outcomes measured were reported as percentages. The percent difference associated with each outcome was converted to a common scale using the effect size index Cohen's *h*, representing the standardized difference between the trained and the untrained TBA group on the outcome of interest. The variance-weighted mean effect size for each sub-group of outcomes was then calculated, and homogeneity tests were performed on the distributions of the weighted mean effect sizes. With a few exceptions, homogeneity of variance was rejected (α=<0.05), and the weighted mean effect size and 95% confidence interval were calculated using formulae based on a random effects model. Sensitivity analysis was conducted to detect the presence of publication bias. In addition, stratified analyses, by study design and sampling procedure, were performed to examine the influence of these potential moderating variables on the weighted mean effect sizes. Lastly, sub-group analyses conducted to date include antenatal care, referral for emergency obstetric care, birthing and newborn-care practices, perinatal and newborn health outcomes.

## RESULTS

The findings of the meta-analysis were positive for the sub-groups of outcomes measured (Table 1). Stratifying each sub-group analysis by the study design and sampling procedure did not change the overall conclusions. The medium-to-large effect size values for behaviour of TBAs relating to intrapartum, including safe delivery, clean delivery, and cord-care practices, represented significant increases of 44%, 103%, and 53% respectively for the trained TBA group over the untrained TBA group at baseline. Likewise, the medium-to-large effect size values for behaviour of TBAs relating to counselling on maternal nutrition, early exclusive breastfeeding, and immunization (primarily TT) represented significant increases (15%). The moderate-to-large effect sizes for knowledge and behaviour of TBAs relating to antenatal care—positive medium-to-large values—were also significant and represented 177% and 47% increases respectively for the trained TBA group over the untrained TBA group at baseline, as was the small effect size for maternal behaviour relating to use of antenatal care services—a 38% increase for women cared for by trained TBAs (14). The small effect sizes for both behaviour of TBAs and maternal behaviour relating to referral of obstetric complications were significant and represented 36% and 22% increases respectively for the trained TBA group over the untrained TBA group at baseline (15). However, the large standard errors and wide confidence intervals revealed the variability in these TBA and maternal attribute data. Lastly, the effect size value for perinatal mortality represented a significant 8% decrease in mortality associated with TBA training (13). Although the number of studies included in the latter sub-analysis was small, the effect size values for birth asphyxia and mortality due to pneumonia represented 11% and 8% decreases respectively in cause-specific newborn mortality (13). Small standard errors and narrow confidence intervals highlighted the homogeneity of these outcome data.

**Table 1.** Summary of findings: meta-analysis of effectiveness of TBA training

Finding	No. of studies reporting outcomes	Pooled treatment group (comparison group)	Effect size mean (SEM)	95% CI	Increase over baseline (%)
TBA behaviour					
Safe delivery	16	3,929 (5,864)	0.35 (0.08)*	0.19–0.51	44
Clean delivery	15	2,566 (4,062)	0.72 (0.12)*	0.49–0.96	103
Cord care	16	2,502 (2,996)	0.41 (0.08)*	0.24–0.57	53
Maternal nutrition	6	905 (842)	0.42 (0.13)*	0.16–0.68	53
Breastfeeding	10	1,170 (1,663)	0.70 (0.17)*	0.37–1.30	100
Immunization	13	1,826 (2,122)	0.55 (0.10)*	0.35–0.74	73
Knowledge of TBA (14–15)					
Referral, antenatal care	3	193 (477)	0.97 (0.29)*	0.40–1.55	177
Referral, obstetric complications	6	441 (786)	0.37 (0.21)	-0.05–0.78	–
Behaviour of TBA (14–15)					
Referral, antenatal care	6	626 (650)	0.39 (0.14)*	0.12–0.67	47
Referral, obstetric complications	13	5,976 (5,991)	0.30 (0.08)*	0.15–0.45	36
Maternal behaviour (14–15)					
Antenatal care-use	10	4,919 (3,368)	0.33 (0.07)*	0.19–0.46	38
Emergency obstetric care-use	2	2,812 (1,567)	0.21 (0.09)*	0.03–0.38	22
Perinatal outcomes (13)					
Overall mortality	17	15,286 (12,786)	0.07 (0.01)*	0.04–0.09	8
Mortality due to birth asphyxia	3	6,217 (5,170)	0.11 (0.05)*	0.02–0.21	11
Mortality due to pneumonia	2	5,333 (4,995)	0.08 (0.02)*	0.04–0.12	8

*p<0.05

CI=Confidence interval;

SEM=Standard error of mean;

TBA=Traditional birth attendant

## DISCUSSION

TBA training is associated with moderate-to-large improvements in behaviours relating to selected intrapartum and postnatal care practices, and small but significant decreases in perinatal mortality and neonatal mortality due to birth asphyxia and pneumonia. The findings are consistent with the historical focus of TBA training on extending the reach of primary healthcare and a few TBA training programmes that have included management of birth asphyxia and pneumonia care. TBA training is also associated with small but significant increases in women's use of antenatal care and emergency obstetric care, suggesting that referral is a multi-faceted process, and influence of TBAs on care-seeking behaviour may be limited.

Unfortunately, incomplete reporting by authors resulted in loss of studies and datasets and limited our ability to examine which intervention characteristics were associated with better outcomes, cost-effectiveness, and the association between training and maternal mortality—the rationale for the policy shift away from TBA training. Moreover, due to the variable quality of studies included in our sample, we could not address causality and only the magnitude and direction of the association between training and the outcomes could be measured. More rigorous studies are now being conducted. In a recent cluster-randomized controlled trial from Pakistan, Jokhio and colleagues showed that TBA training, linked to outreach and facility-based care, resulted in a statistically significant reduction of 30% in perinatal mortality (16). The estimated percent reduction was similar for maternal mortality, but it was not significant.

**Fig. 1.**
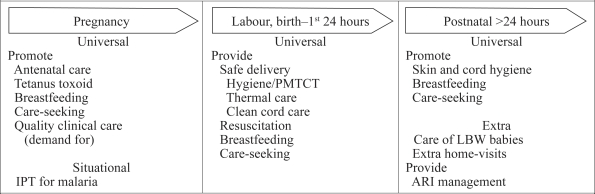
Family and community interventions that reduce newborn mortality while improving maternal health*: what trained TBAs can do

**Fig. 2.**
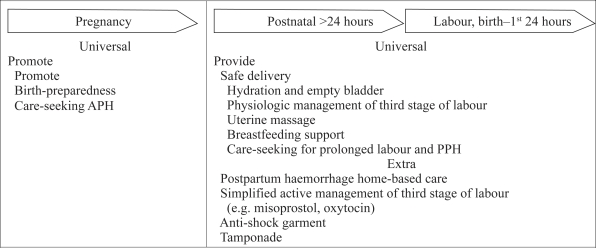
Family and community interventions with potential to reduce maternal mortality due to postpartum haemorrhage: what trained TBAs could do

Millennium Development Goal (MDG) 4 aims to reduce mortality of children, aged less than five years, by two-thirds between 1990 and 2015. MDG 5 aims to reduce maternal mortality by three quarters during this timeframe. Policy regarding the best strategies to meet these goals, however, swings between community-based care and facility-based care, while safe motherhood and child-survival advocates compete for limited resources (2–3). The findings of the meta-ana-lysis suggest that trained TBAs can contribute to MDG 4 through participation in key evidence-based interventions (Fig. 1). For example, at 90% coverage, universal, extra, and situational interventions would result in a reduction of 15–32% in newborn mortality while improving maternal health (1). There are no comparable data for maternal mortality. Empirical evidence nonetheless suggests that hydration, keeping the bladder empty during labour, and stimulating the uterus to contract after delivery reduces postpartum haemorrhage due to uterine atony. Thus, there is an urgent need for research on these and on other community-based interventions that may reduce postpartum haemorrhage, a leading cause of maternal death (Fig. 2).

*Adapted from Darmstadt *et al.*, 2005 (1). Interventions @ 90% coverage result in an estimated 15–32% reduction in newborn mortality
ARI=Acute respiratory infection;
IPT=Intermittent presumptive treatment;
LBW=Low birth-weight;
PMTCT=Prevention of maternal-to-child transmission of HIV

APH=Antepartum haemorrhage;
PPH=Postpartum haemorrhage;
TBA=Traditional birth attendant

In settings characterized by high mortality and weak healthcare systems, family and community care providers often include a mix of women, family members, TBAs, and other semi-skilled or skilled health workers. In these circumstances, “Are TBAs the best choice, among others, for training in health promotion and delivery care?” There are a number of considerations. One important consideration is the proportion of deliveries assisted by TBAs since this reflects, to a large extent, their number, distribution, role, status, presence of other providers, and preference of women and families. Our analysis of the 1994–2004 Demographic and Health Survey data from 44 countries for the period of three years preceding the survey (17) showed that the proportion of deliveries assisted by TBAs is extremely variable within and across countries, being highest for the rural areas. The proportion, however, is relatively low on average and is comparable to that of deliveries assisted by family members and no one combined (Table 2). It is, thus, essential to assess healthcare use-patterns, existing infrastructure, and availability of providers in local, regional and national settings for inputs into policy decision-making. We suggest that, where coverage is high, training TBAs to provide key evidence-based interventions and first-aid care for selected complications is a viable short-term strategy.

**Table 2.** Proportion of deliveries assisted by unskilled attendants

Type of attendant	Residence	Average % (SD)	Range (%)
TBA	Urban	12 (12)	0–50
	Rural	29 (20)	2–79
Family + other	Urban	9 (8)	<1–33
	Rural	27 (17)	<1–62
No one	Urban	2 (2)	0–10
	Rural	5 (5)	<1–22
Unknown	Urban	<1 (<1)	0–1
	Rural	<1 (<1)	0–1

Data analyzed using DHS StatCompiler (17) for 1994–2004

n=44 countries representing three world regions

Percentages based on 62,492 urban births and 144,924 rural births

DHS=Demographic and health survey;

SD=Standard deviation;

TBA=Traditional birth attendant

The authors of “Newborn health: a key to child survival” in a 2005 special issue of *The Lancet*—eloquently argued that women, newborns, and children all have lefts, and all would greatly benefit from a health system that delivers proven interventions through a continuum-of-care approach, i.e. from pregnancy through labour, birth, and the postpartum and postnatal periods, and into early childhood; and including home, peripheral health facility, and hospital (1, 18). They contended that, with sufficient coverage, affordable low-tech approaches could reduce the number of newborn deaths by up to one-third and prevent over half of child deaths. Thus, they urge a balanced, phased approach to policy and programming such that service modalities of family-community care and outreach save lives now, especially among the poor, while the health system is simultaneously strengthened, and facility-based clinical care is made available and equitable, in time. To achieve even greater reductions in newborn, and early child mortality and to reduce maternal mortality, they emphasized that facility-based clinical care, including skilled birth attendance and an intact referral system, i.e. a functioning health system, is required.

Given an environment of scarce resources, there is an understandable concern among safe motherhood advocates that investment in family-community care approaches will set-back advances made with respect to increasing skilled birth attendance and emergency obstetric care. We agree with the Millennium Task Force on Child Health and Maternal Health that, “A strategy designed to address maternal mortality as its true aim—and not just some welcome, but coincidental by product of a health intervention designed primarily for another purpose (averting newborn death, for example)—must include interventions that prevent and treat complications that kill women” (2). We believe that a balanced, phased approach encompasses such interventions and that, realistically, there is no ethical alternative. To improve pregnancy outcomes, there is a critical need, not only to upgrade health facilities and train, strategically deploy, and retain professional care providers, but also to evaluate, refine, and disseminate promising community-based approaches to care during birth and the immediate postpartum and postnatal periods.

## ACKNOWLEDGEMENTS

The meta-analysis of effectiveness of TBA training was made possible through funding support from the World Bank (Small Grants Program for Safe Motherhood), USAID (Intra Health/PRIME I Project, Academy for Educational Development/SARA Project), USAID, DFID and the Bill and Melinda Gates Foundation (University of Aberdeen/IMMPACT Project), and the Bill and Melinda Gates Foundation (Save the Children/Saving Newborn Lives Project). The views presented here represent those of the authors.
